# Two Arginine Residues Suppress the Flexibility of Nucleosomal DNA in the Canonical Nucleosome Core

**DOI:** 10.1371/journal.pone.0120635

**Published:** 2015-03-18

**Authors:** Hidetoshi Kono, Kazuyoshi Shirayama, Yasuhiro Arimura, Hiroaki Tachiwana, Hitoshi Kurumizaka

**Affiliations:** 1 Molecular Modeling and Simulation, Japan Atomic Energy Agency, 8-1-7 Kizugawa, Kyoto 619-0215, Japan; 2 Laboratory of Structural Biology, Graduate School of Advanced Science and Engineering, Waseda University, 2-2 Wakamatsu-cho, Shinjuku-ku, Tokyo 162-8480, Japan; Jacobs University Bremen, GERMANY

## Abstract

The dynamics of nucleosomes containing either canonical H3 or its centromere-specific variant CENP-A were investigated using molecular dynamics simulations. The simulations showed that the histone cores were structurally stable during simulation periods of 100 ns and 50 ns, while DNA was highly flexible at the entry and exit regions and partially dissociated from the histone core. In particular, approximately 20–25 bp of DNA at the entry and exit regions of the CENP-A nucleosome exhibited larger fluctuations than DNA at the entry and exit regions of the H3 nucleosome. Our detailed analysis clarified that this difference in dynamics was attributable to a difference in two basic amino acids in the αN helix; two arginine (Arg) residues in H3 were substituted by lysine (Lys) residues at the corresponding sites in CENP-A. The difference in the ability to form hydrogen bonds with DNA of these two residues regulated the flexibility of nucleosomal DNA at the entry and exit regions. Our exonuclease III assay consistently revealed that replacement of these two Arg residues in the H3 nucleosome by Lys enhanced endonuclease susceptibility, suggesting that the DNA ends of the CENP-A nucleosome are more flexible than those of the H3 nucleosome. This difference in the dynamics between the two types of nucleosomes may be important for forming higher order structures in different phases.

## Introduction

Eukaryotic cells compactly organize their large genomes in the form of chromatin. Chromatin is constructed of repeats of nucleosomes and linker DNA. The nucleosome is the fundamental structural unit, comprising a histone octamer formed by two copies each of the four core histones H3, H4, H2A, and H2B and approximately 150 bp of DNA that wraps around the octamer. There are variants of these histone proteins. In humans, the variants include H3.1 (canonical), H3.2, H3.3, H3T, H3.5, H3.X, H3.Y, and CENP-A [1], each of which is likely to have a specific role. In cells, chromatin comprises mixtures of different types of nucleosomes. Thus, the experimentally observed characteristics of chromatin represent an average view. To understand how the dynamics of chromatin depend on the types of nucleosomes, it is essential to characterize the physicochemical properties of individual nucleosomes or the fundamental structural unit of chromatin.

Among H3 variants, CENP-A has been greatly investigated because the CENP-A-containing nucleosome (hereafter, the CENP-A nucleosome) provides key epigenetic information that marks locations of centromeres by dictating centromeres in the chromosome [2]. The crystal structure of the CENP-A nucleosome has been determined recently to be composed of a histone octamer [3], although there is controversy regarding the histone composition and stoichiometry of the CENP-A nucleosome [4]. Similar to the canonical H3 nucleosome, the crystal structure of the CENP-A nucleosome is composed of two sets of H2A, H2B, CENP-A (instead of H3), and H4 and nucleosomal DNA. However, the structures of the entry and exit regions of DNA are not visible in X-ray crystallographic analysis, thereby suggesting that the DNA termini are flexible [3]. Panchenko et al. [5] have also shown that flanking DNA is more flexible in a CENP-A nucleosome than in the canonical nucleosome, and they have speculated the cause from an H3 nucleosome structure. However, the mechanism is not well understood.

In the present study, we performed several molecular dynamics (MD) simulations and investigated the cause of suppressed flexibility in the canonical nucleosome. The results suggest that the main reason for the suppression is the higher affinity of two arginine (Arg) residues in the αN helix of H3 for DNA compared with that of two lysine (Lys) residues at the corresponding sites in CENP-A. In addition, we propose a mechanism for the control of flexibility. To the best of our knowledge, this is the first study to use MD simulations to compare the dynamics of noncanonical and canonical nucleosomes [6].

To experimentally confirm the MD results, two Arg residues in H3 were mutated into Lys residues, and a nuclease digestion assay was performed to determine whether the endonuclease susceptibility of nucleosomal DNA changed. The results demonstrate that the susceptibility of the nucleosome containing H3 mutants (R49K and R53K) increased and was comparable with that of the CENP-A nucleosome. However, the nucleosome with opposite mutations in CENP-A, in which two Lys residues were mutated into Arg residues, was still susceptible to the endonuclease. This finding suggests that the two Arg residues in H3 are responsible for stabilizing the nucleosome structure and the tight wrapping of nucleosomal DNA, although the instability of the CENP-A nucleosome is not fully accounted for by these residues. Based on the structure of the CENP-A nucleosome, it appears that a bulky residue, Trp46, adjacent to the αN helix prevents favorable interactions between the mutated Arg residues and DNA.

## Methods

### Atomic models

To compare the stability of nucleosomes containing the canonical H3 histone and its variant CENP-A, four independent MD simulations were performed (Table 1). The simulations were performed with two crystal structures, i.e., the canonical H3 nucleosome (PDB code: 1kx5)[7] and the CENP-A nucleosome (PDB code: 3an2) [3] and two structures in which the nucleosomal DNA sequences were exchanged to determine whether DNA sequence dependency occurred, although both DNAs were α-satellite derivatives. DNA structures at both ends of nucleosomal DNA (13 bp at each end) were missing from the CENP-A nucleosome, but they were modeled based on the DNA structure of the canonical H3 nucleosome by structurally aligning the phosphate atoms of backbones (residue numbers, from −60 to 60) of 3an2 with those of 1kx5 to ensure that the length of DNA was equal for all systems. The missing DNA of the CENP-A nucleosome was modeled using the same conformational parameters as those of the corresponding DNA of the H3 nucleosome using X3DNA [8]. The histone tails of 1kx5 were truncated to adjust the length of the corresponding histones of 3an2, thereby ensuring that the simulation systems were as similar as possible. Finally, residues from 46 to 132 in H3, 25 to the C-terminal in H4, 16 to 114 in H2A, and 32 to 121 in H2B were used for MD simulations. The effect of the histone tails, likely to be of significance, will be the subject of a future investigation.

**Table 1:** Simulation systems.

name	Histone 3	DNA seq.	Length	Structure
**CENP-A-NCP**	CENP-A	α-satellite based sequence, from 3an2	100ns	3an2
**CENP-A-NCPsw**	CENP-A	α-satellite based sequence from 1kx5	50ns	DNA from 1kx5Histones from 3an2
**H3-NCP**	Canonical H3	α-satellite based sequence from 1kx5	100ns	1kx5
**H3-NCPsw**	Canonical H3	α-satellite based sequence from 3an2	50ns	DNA from 3an2, Histones from 1kx5

### Simulation protocol

All four nucleosomes were subjected to the same MD protocol. Only coordinates of proteins and DNA were derived from the crystal structures because the crystal structure of the CENP-A nucleosome did not have the coordinates for water and ions. First, TIP3P [9] water molecules were added so the thickness was at least 20Å from the surface of the nucleosome (an approximately 150 × 150 × 150 Å^3^ box). Sodium ions were added to neutralize each system (final salt concentration, approximately 120 mM). The total number of atoms was approximately 315,000 for each system, and all interactions in each system were considered in the simulations. MD simulations were performed using the SANDER module of the AMBER 10 package [10] with the following conditions: the force fields used were ff99SB for protein [11], bsc0 for DNA [12], and ions08 for ions [13]; the time step was 2 fs, and all bonds containing hydrogen were frozen with the SHAKE algorithm [14]; the temperature (NPT) was regulated at 300 K with Langevin dynamics with a collision frequency of 2.0 ps^−1^ for each atom; the pressure was regulated at 1 atm using a weak coupling algorithm with a relaxation time of 1.0 ps [15]; electrostatic interactions were calculated with the particle mesh Ewald method [16]; and van der Waals interactions were calculated with a cutoff of 9.0 Å.

First, the systems were minimized on the positions of the hydrogen atoms for 1000 cycles, followed by minimization of the entire system for 10,000 cycles. Next 100-ps MD simulations were performed at 10 K with a 10 kcal/mol positional restraint for all atoms, except water molecules and ions, followed by seven 100-ps MD simulations at 300 K with the gradual release of positional restraints for all atoms, except water molecules and ions, as follows: 5, 2, 1, 0.5, 0.2, 0.1, and 0.0 kcal/mol. Finally, 50- to 100-ns product runs were performed for each system. The trajectories were analyzed using the ptraj program of the AMBER package [17] and in-house tools.

### Exonuclease III assay

The susceptibility of nucleosomal DNA to exonuclease III (ExoIII) was assessed. Four types of nucleosomes were prepared: H3.1, CENP-A, H3.1 with R49K and R53K mutations, and CENP-A with K49R and K53R mutations. The nucleosomes were reconstituted using a salt dialysis method with the human recombinant histones H2A, H2B, H4, and H3.1, CENP-A, H3.1 with R49K and R53K mutations, or CENP-A with K49R and K53R, as described previously [3, 18]. These reconstituted nucleosomes were purified by native polyacrylamide gel electrophoresis using a Prep Cell apparatus (Bio-Rad). Solutions containing nucleosomes (40 ng of DNA/μL) and ExoIII (200 mU/μL) were incubated in 10 μL of 63 mM Tris-HCl buffer (pH 8.0) containing 5 mM KCl, 5 mM MgCl_2_, and 2.5 mM dithiothreitol at 37°C for 2.5, 5, and 10 min, and these solutions were deproteinized by phenol/chloroform extraction and ethanol precipitation, followed by denaturing gel electrophoresis (10% PAGE with 7 M urea). DNA was visualized by ethidium bromide staining. Note that in the ExoIII assay, the amino acid sequence of H3.1 was used instead of H3.2, which was used in the simulations. H3.1 only differs from H3.2 at positions 96 and 102; H3.1 contains cysteine (Cys) and glycine (Gly), whereas H3.2 contains serine (Ser) and alanine (Ala). These residues are located at positions where they do not interact directly with DNA, and the amino acid differences are expected to have little effect on the dynamics of nucleosomal DNA.

## Results

To determine whether nucleosomal dynamics depend on DNA sequences, we performed two 100-ns simulations of the original combinations of histones and nucleosomal DNA (which were solved by X-ray crystallography and registered in the Protein Data Bank as 1kx5 and 3an2), and we performed two 50-ns simulations of the systems in which the nucleosomal DNA sequences were exchanged between 1kx5 and 3an2. We refer to these four systems as H3-nucleosome core particle (NCP), CENP-A-NCP, H3-NCPsw, and CENP-A-NCPsw (Table 1). Histone tails were not visible in the X-ray crystal structures of the nucleosomes. Therefore, in the simulations, tail truncation was performed so the simulation systems containing either canonical histone H3 or its variant CENP-A were as similar as possible. We cannot exclude the possibility that the tail truncation might exaggerate the dynamics of nucleosomes. However, we believe that there are differences in dynamics between H3- and CENP-A nucleosomes that are observed in the simulations, because the biochemical data from ExoIII assays (described later) consistently support the differences.

### Structural fluctuations were independent of the DNA sequence but dependent on histone proteins

**Fig 1:**
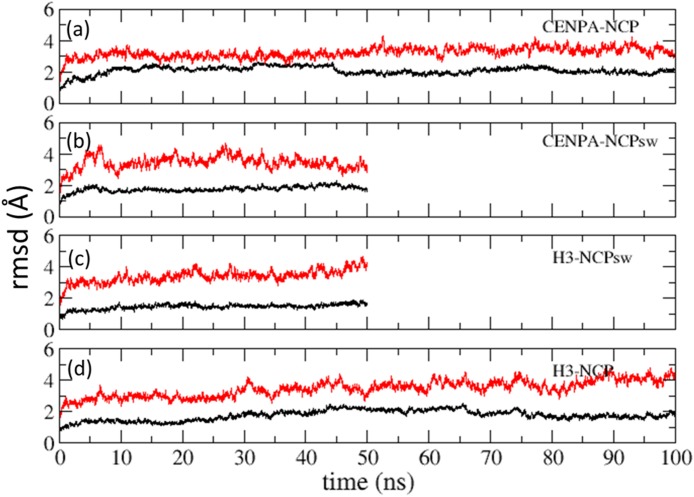
Root mean square deviations (rmsds) of nucleosomes. Rmsds were calculated using Cα atoms for histones (black lines) and P atoms for DNA excluding 20bp from either of ends (red lines). (a) CENP-A-NCP (b) CENP-A-NCPsw (c) H3-NCPsw and (d) H3-NCP.

The root mean square deviation (rmsd) was calculated with Cα atoms for histones and P atoms for DNA, excluding 20 bp from each end. The rmsd values showed that the histone cores of all four nucleosomes and DNA were stable (standard deviations, 0.2–0.4 Å), irrespective of differences in the constituent histones and the DNA sequences (Fig. 1). Fluctuations in the rmsd of the CENP-A nucleosome, CENP-A-NCP, and CENP-A-NCPsw were comparable with those in the rmsd of the H3-containing nucleosomes, H3-NCP, and H3-NCPsw, indicating that fluctuations were not sensitive to the initial DNA models.

**Fig 2:**
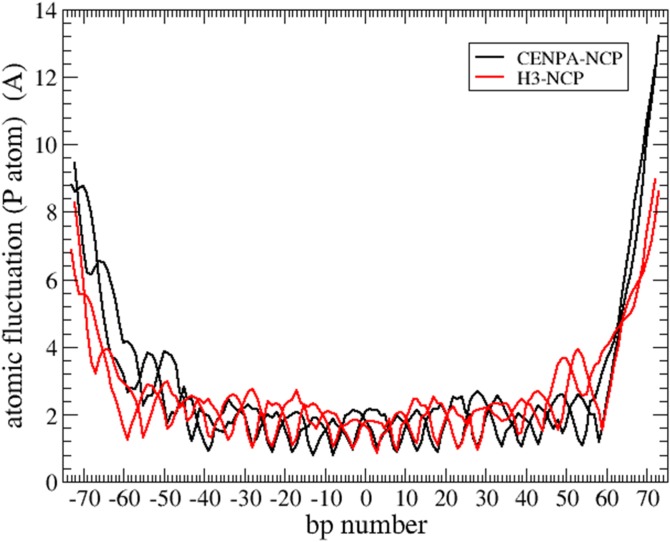
Atomic fluctuation of P atoms in DNA. The fluctuations were calculated using 100-ns long trajectories. CENP-A-NCP (black) and H3-NCP (red). Two lines for each of nucleosomes denote two DNA chains.

We observed large fluctuation in DNA at the entry and exit regions in both CENP-A and H3-containing nucleosomes. The atomic fluctuations of P atoms were large at both ends of nucleosomal DNA, whereas the remaining sequence was wrapped tightly around the histone core (Fig. 2). The ends of DNA maintained base pairing despite large fluctuations. Fluctuations other than those at the ends had a periodicity of 10 bp, irrespective of the histone component and the DNA sequence. This result indicates that the similar dynamics of H3-NCP and CENP-A-NCP are attributable to protein–DNA interactions. At the ends of DNA, the atomic fluctuations of CENP-A-NCP were much larger than those of H3-NCP, indicating that DNA in CENP-A-NCP was unwrapped more than that in H3-NCP. It is notable that fluctuations started to increase from the bp number of −45 or +45 to −73 or +73 at the ends in both H3-NCP and CENP-A-NCP, indicating that the unwrapping effect extended up to 28 bp from the end of DNA.

### Distribution of the distance between the end of DNA and the center of mass of the histone core

**Fig 3:**
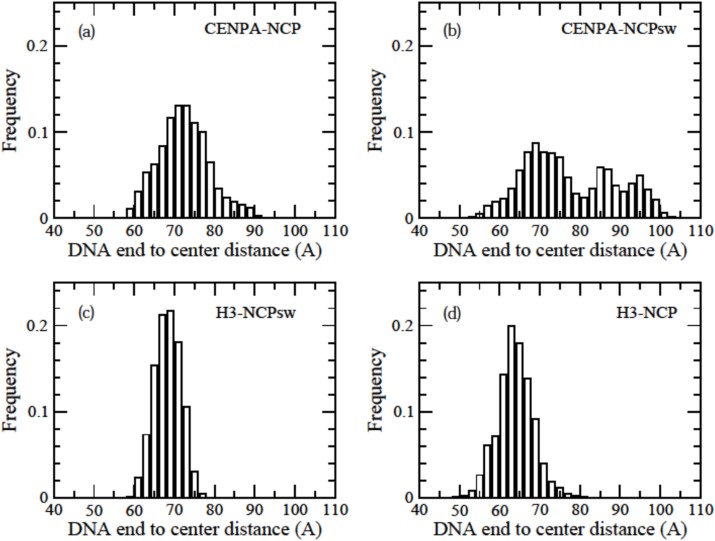
Distributions of the distances between the ends of the DNA sequence and the center of mass of the histone core. Frequencies were normalized by number of sampling. (a) CENP-A-NCP, (b) CENP-A-NCPsw, (c) H3-NCPsw and (d) H3-NCP.

**Fig 4:**
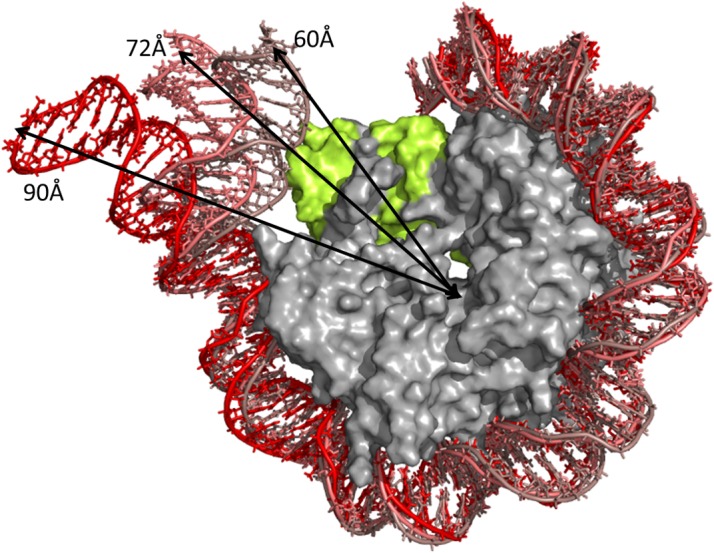
Snap shots from CENP-A-NCP trajectory were superimposed on the histone core. Three conformations with distances between DNA end and the center of mass of histone core of 60, 72 and 90Å (represented by arrows) are shown. CENP-A is colored by yellow and a half of DNA is shown for clarity. The figure was prepared using PyMol (The PyMOL Molecular Graphics System, Schrödinger, LLC).

The distance was measured between the N1 atom of the 5’ base and the center of mass of the histone core considering all constituting atoms. Fig. 3 shows the distribution of the distance. The average distances (±standard deviations) observed in MD simulations were 72.4 ± 6.3 Å for CENP-A-NCP, 77.5 ± 11.3 Å for CENP-A-NCPsw, 68.3 ± 3.2 Å for H3-NCPsw, and 63.9 ± 4.4 Å for H3-NCP, which were much larger than the distances obtained from the H3-NCP crystal structure (57.7 and 56.0 Å for the two ends). Even in H3-NCP, the average distance was at least 8 Å longer than that obtained from the crystal structure. Fig. 3 clearly shows that DNA in CENP-A-NCP and CENP-A-NCPsw had broad distributions. In contrast, there was a clear peak for H3-NCP and H3-NCPsw. These results indicate that the entry and exit regions of DNA in the CENP-A nucleosome unwind and rewrap with lower energy costs than the H3 nucleosome. It is notable that CENP-A-NCPsw had two or three peaks, because one end of DNA was never rewrapped once it was unwrapped. The other three nucleosomes exhibited unwrapping and rewrapping, producing gauss-shaped distributions (Fig. 3). Fig. 4 shows three typical snapshots from the CENP-A-NCP trajectory with DNA end-to-center distances of 60, 72, and 90 Å. When DNA was wrapped tightly around the histone core and interacted with CENP-A, the distance was approximately 60 Å. We observed that 10–15 bp from the end of DNA were unwrapped at a distance of approximately 70 Å, where part of the CENP-A–DNA interaction was lost, but H2A–DNA and H2B–DNA interactions were maintained. Such conformations were also observed in the other three nucleosomes. Furthermore, we observed that DNA could bend toward the major groove at 6 bp from the end, producing a large distance of 90 Å.

### Key residues responsible for wrapping nucleosomal DNA

The cause of the difference in fluctuations in DNA between the H3- and CENP-A-NCPs is unknown. Amino acid sequence comparison showed that two positively charged Arg residues in the αN helix of H3 were replaced by Lys residues at the corresponding sites in CENP-A (see Fig. 2A in reference [3]). Our detailed observations of interactions between the amino acids and DNA showed that an Arg49 residue in H3-NCPs generally maintained hydrogen bonds with the phosphate backbone of nucleosomal DNA, whereas the corresponding Lys residue in the αN helix of the CENP-A readily lost these bonds (See S1 and S2 Movies).

We calculated the total number of hydrogen bonds between DNA and residues 49, 53, and 56 within every 1 ns (Table 2). The numbers were averaged over 100 ns for CENP-A-NCP and H3-NCP and over 50 ns for CENP-A-NCPsw and H3-NCPsw. In both CENP-A-NCP and H3-NCPsw, these residues did not always form hydrogen bonds. However, H3-NCPs had significantly more hydrogen bonds (2.7–3.5) than CENP-A-NCPsw (0.4–0.8). S1 Fig. shows the rate of hydrogen bond formation by residues 49, 53, and 56 during each 1-ns interval of the simulation period. After hydrogen bonds were lost by Lys residues in CENP-A, the end part (approximately 15 bp) of DNA preferentially remained in the solvent and was not readily rewrapped during the simulation period of 100 ns. Of the three positively charged residues, Arg49 appeared to play a critical role in keeping DNA wrapped. During the 100-ns simulation period, we observed that Arg49 almost always maintained hydrogen bonds by changing the hydrogen bond partner atoms (see S1 and S2 Movies).

**Table 2:** Time-averaged number of hydrogen bonds between DNA and residues 49, 53 and 56^a^.

MD system	Number of hydrogen bonds
**CENP-A-NCPM**	0.8 (0.4)
**CENP-A-NCPsw**	0.4 (0.3)
**H3-NCP**	3.5 (1.2)
**H3-NCPsw**	2.7 (0.8)

^a^Average was calculated every 1ns. The values in parenthesis are standard deviation over 100ns for CENP-A-NCP and H3-NCP and 50ns for CENP-A-NCPsw and H3-NCPsw, respectively.

### Dynamics of histone cores

**Fig 5:**
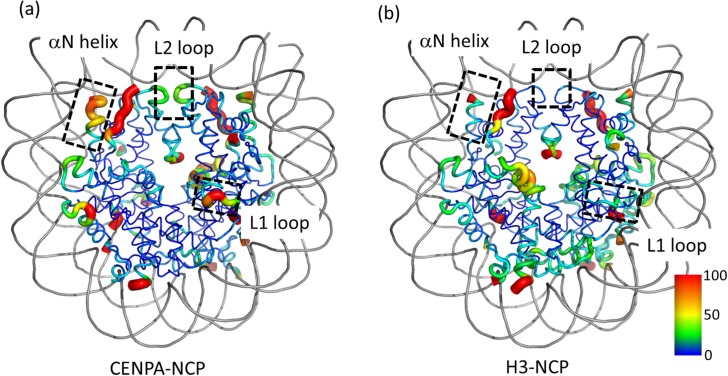
Atomic fluctuations of NCPs. (a) CENP-A-NCP and (b) H3-NCP. The B-factor values of the main chain heavy atoms calculated using 100 ns MD trajectories are mapped onto the structures in different colors, where lower values are denoted in blue and higher values in red. Residues with higher B-factor values are also denoted by tubes with a greater radius. The figures were prepared using PyMol (The PyMOL Molecular Graphics System, Schrödinger, LLC).

Fig. 5 shows the atomic fluctuations of the main chain heavy atoms of the histone cores. In both CENP-A-NCP and H3-NCP, fluctuations in the core region were small (shown in blue), and fluctuations in the N- and C-termini of histones were large (shown in red). However, some remarkable differences were observed. In CENP-A-NCP, there were large fluctuations in the αN helix and the L1 and L2 loops. In contrast, the corresponding regions in H3-NCP exhibited smaller fluctuations. The αN helix was located at the entry and exit regions of DNA. As expected, because these parts of DNA in CENP-A-NCP were unwrapped more than those in H3-NCP, the αN helix showed large fluctuation. The L1 loop of CENP-A is longer than that of H3 by two residues and is protruding into the solvent [3]. This difference in length also produces large fluctuation. The L2 loop is located at the dyad of DNA (0 bp in bp number). Fluctuations of DNA around the dyad in H3-NCP were smaller than those in CENP-A-NCP (Fig. 2), which was consistent with smaller fluctuations in the L2 loop of H3-NCP compared with those in the L2 loop of CENP-A-NCP. This difference was also attributed to differences in the DNA dynamics of H3-NCP and CENP-A-NCP.

### Dynamics of the CENP-A targeting domain (CATD)

**Fig 6:**
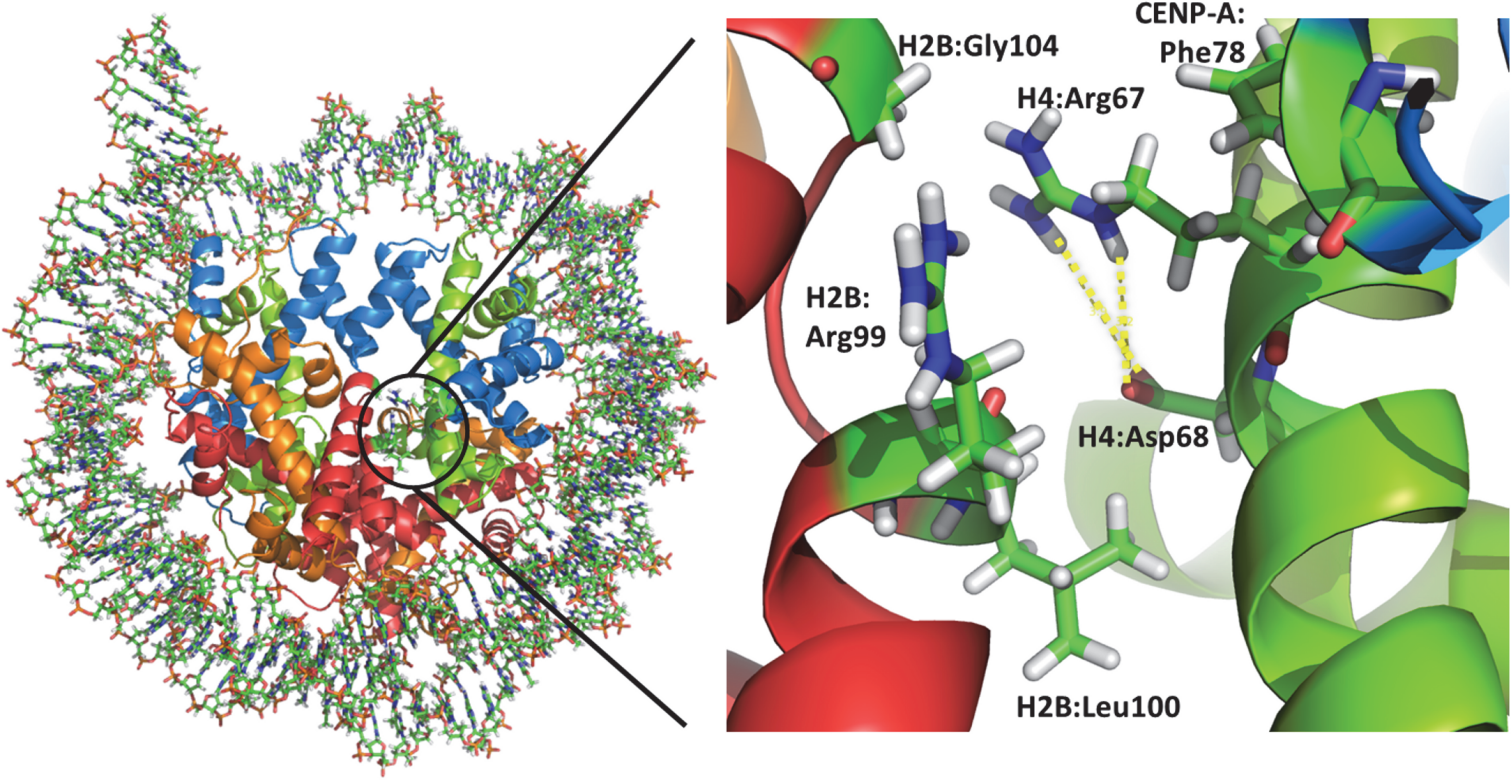
A close up view around Arg67 of H4 in CENP-A-NCP.

CENP-A contains the CENP-A targeting domain (CATD; residues 75–114) and this domain is considered to rigidify the CENP-A–H4 interface (residues 54–81) because of its stronger hydrophobic interaction compared with H3-NCP [19]. Our MD simulations showed relatively small fluctuations in main chain atoms of these regions in both CENP-A-NCP and H3-NCP, except for the L1 loop in CENP-A (Fig. 6 and S2 Fig.). However, a remarkable difference was detected when we focused on fluctuations in side chains (S2 Fig.). Fluctuations in the side chains of H4 in CENP-A-NCP were suppressed, indicating tight packing in the interface. The side chain of Arg67 of H4 in H3-NCP fluctuated greatly compared with the side chain of the corresponding residue in CENP-A-NCP. Detailed observations showed that the side chain of Arg67 in CENP-A-NCP had favorable interactions with the main chains of Arg99, leucine (Leu) 100, and Gly104 in H2B, the side chain of aspartic acid (Asp) 68 in H4, and phenylalanine (Phe) 78 in CENP-A (Fig. 6). These interactions were not observed in H3. This result indicates that local differences in the amino acid sequence in this region affect the packing of H2B and H4, although CENP-A-NCP and H3-NCP both contain the same H2B and H4.

The structure is a snap shot derived from CENP-A-NCP trajectory. In carton model, CENP-A, H4, H2A and H2B are colored by blue, green, orange and red, respectively. In stick model, carbon, nitrogen, oxygen and hydrogen are colored by green, blue, red and white, respectively. The figures were prepared using PyMol (The PyMOL Molecular Graphics System, Schrödinger, LLC).

In CATD, Arg83 in H3 was substituted by asparagine (Asn). A previous X-ray crystal structural analysis of H3-NCP [20] suggested that the insertion of Arg83 in H3 in the minor groove of nucleosomal DNA stabilized NCP. Atomic fluctuations obtained in the present study did not show any difference between CENP-A-NCP and H3-NCP at this residue site. During the simulation period of 100 ns, Arg83 in H3 remained inserted in the minor groove, and fluctuation was small, while fluctuation in Asn85 in CENP-A was also small despite the lack of the insertion. This small fluctuation occurred because Asn85 in CENP-A was located in the vicinity of the minor groove, and it had a favorable interaction with the DNA phosphate backbone.

### Exonuclease susceptibility assay

**Fig 7:**
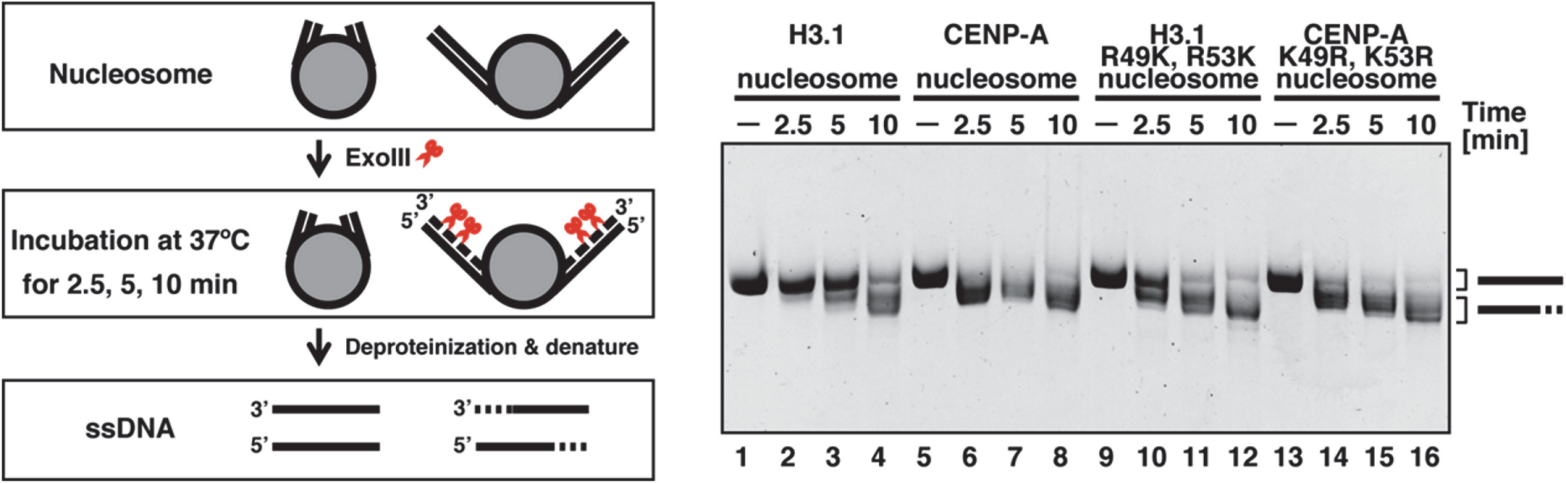
Exonuclease (ExoIII) susceptibility of the nucleosomes containing CENP-A and H3 mutants. Lanes 1–4, lanes 5–8, lanes 9–12, and lanes 13–16 represent experiments performed with canonical H3, CENP-A, H3 mutant (with R49K and R53K), and CENP-A mutant (with K49R and K53R), respectively. The reaction periods are 0 min (lanes 1, 5, 9, and 13), 2.5 min (lanes 2, 6, 10, and 14), 5 min (lanes 3, 7, 11, and 15), and 10 min (lanes 4, 8, 12, and 16).

MD simulations suggested that two Arg residues, Arg49 and Arg53, were key residues suppressing DNA fluctuations by forming more hydrogen bonds with DNA phosphate groups than Lys residues at corresponding positions in CENP-A. Therefore, we performed ExoIII susceptibility assays for the nucleosomes that contained H3 and CENP-A mutants. In canonical H3, Arg49 and Arg53 were mutated into Lys, whereas Lys49 and Lys53 were mutated into Arg in CENP-A. Consistent with the simulation results, the DNA ends of CENP-A-NCP were more susceptible to ExoIII, compared with those of H3-NCP (Fig. 7, lanes 1–8). Interestingly, NCP containing the H3 mutants also exhibited similar patterns of ExoIII susceptibility to CENP-A-NCP, in which DNA degradation began as early as 2.5 min (Fig. 7, lanes 9–12). In contrast, DNA degradation in H3-NCP only began after 5 min of incubation (Fig. 7, lanes 1–4). This finding suggests that these two Arg residues are responsible for the stability of the nucleosomal DNA ends in nucleosomes containing H3. However, these two Lys residues might not be sufficient to induce DNA end flexibility in the CENP-A nucleosome, because mutations of two Lys residues to Arg in CENP-A still exhibited a similar susceptibility pattern to wild type CENP-A (Fig. 7, lanes 13–16).

## Discussion

### Roles of positively charged residues, Arg and Lys

MD simulations performed in this study showed that Arg stabilized nucleosomes more strongly than Lys. Indeed, analysis of protein–DNA interactions based on the complex structures showed that, among 20 amino acids, Arg was the most frequent residue detected in phosphate–amino acid side chain interactions [21]. Arg residues are more likely to form hydrogen bonds than Lys residues, because Arg contains a guanidinium group that has a 2-fold probability of forming hydrogen bonds with the phosphate backbone as compared with the amine group of Lys. In addition, Arg has been shown to have another role in the protein–DNA complex. DNA shape analysis suggests that stretches of A and T bases can narrow the minor groove of DNA, thereby creating binding sites for appropriately placed, positively charged Arg residues and enhancing the local negative electrostatic potential [22]. Lys and Arg are both positively charged residues. However, these results clearly demonstrate a difference in the formation of hydrogen bonds by Lys and Arg, indicating that they have different roles in the protein–DNA interaction.

The strong preference for Arg over Lys is also found in protamines, which are highly positively charged peptides that enable DNA compaction in vertebrate sperm nuclei [23, 24]. Using the osmotic stress technique coupled with X-ray scattering, DeRouchey et al. [25] demonstrated that Arg peptides show a stronger attraction force and weaker short-ranged repulsion than Lys peptides in DNA condensation. However, it is difficult to capture an atomic view from this experiment. DeRouchey et al. [23] proposed that the difference between the DNA–DNA forces with Arg and Lys peptides was the result of a difference in details in the binding mode in DNA grooves.

Recently, a simulation study to investigate the role of the H4 tail in DNA–DNA interactions showed that both Lys and Arg residues played major roles in DNA-DNA attraction by forming bridges and by coordinating to the phosphate groups and to the electronegative sites in the DNA minor groove [26]. In this study, the occupancy of the Arg guanidinium group in the vicinity of phosphate groups was higher than that of the charged group of Lys, whereas the residence time of Arg was shorter than that of Lys. This is consistent with our observation that the guanidinium group has 2-fold probability of forming hydrogen bonds by dynamically exchanging the hydrogen bond partner of NH1 and NH2 atoms. This previous study also provided an atomic view that can explain why Arg residues reside more frequently at the minor groove sites than Lys residues [26]. In the present study, interactions with the electronegative sites in the DNA minor groove of the entry and exit regions were rarely observed, probably because of the truncation of histone tails in our models, thereby enhancing fluctuation in DNA. Another possible explanation for the difference is that the H4 tail simulations used 8 amino acid residue-long fragments (a part of the tail) that were allowed to move freely, which might artificially enhance the occupation of the peptides in DNA grooves.

### Mechanism of stabilization of the histone–DNA interaction

Previously, it was reported that the R49K mutation in H3 decreased the stability of the mononucleosome, possibly by weakening the interaction between the αN helix of CENP-A and DNA [27]. Using hydrogen–deuterium exchange coupled to mass spectrometry, Panchenko et al. [5] showed that DNA at the entry and exit regions of the CENP-A nucleosome was associated more weakly with CENP-A than canonical H3. Furthermore, they showed that the key amino acid involved in this behavior was Lys49 in CENP-A, for which the corresponding residue in H3 was Arg. They reported that the R49K mutation in H3 increased the local flexibility, but the extent of flexibility was not as much as that of CENP-A [5].

Our simulation results that Arg49 is the key residue agree with those obtained by Panchenko et al. [5]. In addition, we showed that Arg53 also contributed to the stability of nucleosomal DNA at the entry and exit regions. However, the mechanism responsible for this stabilization was different. Based on the crystal structure, Panchenko et al. [5] explained that the insertion of Arg49 into the minor groove was important, but the insertion was not maintained during our MD simulations. Instead, the results of our MD study suggest that Arg49 and Arg53 both stabilize protein–DNA interactions by forming more stable hydrogen bonds with DNA than Lys (see S1 and S2 Movies). This may be attributable to differences in Arg and Lys, because an Arg residue is potentially more likely to form hydrogen bonds with the DNA phosphate backbone via its guanidinium group. Thus, we attempted to increase the stability of the CENP-A nucleosome by mutating Lys to Arg. However, the mutation did not restrict the nuclease susceptibility of the DNA ends of CENP-A-NCP. This may be attributable to the amino acid sequences around the αN helix. The αN helix of CENP-A is shorter than that of H3. CENP-A contains Gly46-tryptophan (Trp) 47 before the αN helix. We speculate that this sequence will prevent stable αN helix formation, thereby resulting in inappropriate location of the replaced Arg residues. Alternatively, Arg residues may fail to form hydrogen bonds with DNA because of the spatial hindrance of Trp47.

### Comparison with other MD studies

All atomic simulations of nucleosomes previously reported [28–35], except one in which DNA was intentionally unwrapped by steered MD [30], showed that the DNA superhelix is generally stable, irrespective of the exclusion of histone tails, and did not show any partial unwrapping of DNA observed in the present study. Biswas et al. [29] studied the role of histone tails in structural stability and reported an approximately 20% increase in the rmsd of nucleosomal DNA compared with the simulation of an intact nucleosome. In contrast, we detected large fluctuations in nucleosomal DNA, irrespective of H3 and CENP-A (Fig. 2). We consider that three factors may have caused these differences.

The first factor is the simulation period. In previous studies of nucleosomes lacking histone tails, the calculation periods were 10–50 ns [28, 32], which were shorter than those in the present study. The second factor is the amount of solvent. To reduce the calculation costs in previous studies, the solvent layer that separates the nucleosome from the periodic boundaries was set as short as 9–15 Å [28–35]. In the present study, we included a thicker layer of 20 Å, which thereby provided sufficient space for DNA to fluctuate. Furthermore, it is possible that the layers of 9–15 Å may be too thin, and that they could suppress fluctuation caused by electrostatic interactions with adjacent images when using a periodic condition. The final factor is the length of the truncated tails. We truncated the histone tails to maximize consistency between the two systems of CENP-A-NCP and H3-NCP. However, the resulting truncations were longer than those in previous studies [28, 29, 31]. For example, the H3 tail was 7–19 residues shorter compared with those used in previous studies. A previous Förster resonance energy transfer (FRET) study also suggested that H3 tail truncation (deletion of 1–37 residues) causes nucleosomal DNA ends to largely dissociate from the histone core [36].

## Conclusion

We investigated the dynamics of two distinct nucleosomes, H3 and CENP-A. The results indicate that 100 ns-long simulations are sufficient to observe differences in DNA wrapping. We hypothesized that the substitution of Arg with Lys would reduce the stability of the nucleosome, and that hypothesis was confirmed experimentally. In addition, we proposed a mechanism for the stabilization of nucleosomal DNA by Arg residues.

## Supporting Information

S1 Fig Rate of hydrogen bonds between the histone H3/CENP-A and DNA every 1 ns. The plots are shown for Lys49, Lys53 and Lys56 in CENP-A from CENP-A-NCP and Arg49, Arg53, and Arg56 in H3 from H3-NCP. (a) During the first 50 ns and (b) during the last 50 ns. Note that only protein atoms that form hydrogen bonds with DNA atoms are listed.
(TIF)

S2 Fig Atomic fluctuations of histone proteins. (a) CENP-A-NCP and (b) H3-NCP. The B-factors values of the main chain heavy atoms (black lines) and all heavy atoms (brown lines) were calculated using 100 ns-long MD trajectories. At top of each panel, the secondary structures are shown based on the crystal structures.
(TIF)

S1 Movie H3-DNA interactions during 50 ns. Arg49 and Arg53 of H3 are highlighted by space-fill representation.
(MPG)

S2 Movie CENP-A-DNA interactions during 50 ns. Lys49 and Lys53 of CENP-A are highlighted by space-fill representation.
(MPG)
