# Supplementary figures and images for: Two Arginine Residues Suppress the Flexibility of Nucleosomal DNA in the Canonical Nucleosome Core
Source: PLoS One. 2015 Mar 18;10(3):e0120635. doi: 10.1371/journal.pone.0120635 (PMC4365049; doi:10.1371/journal.pone.0120635)

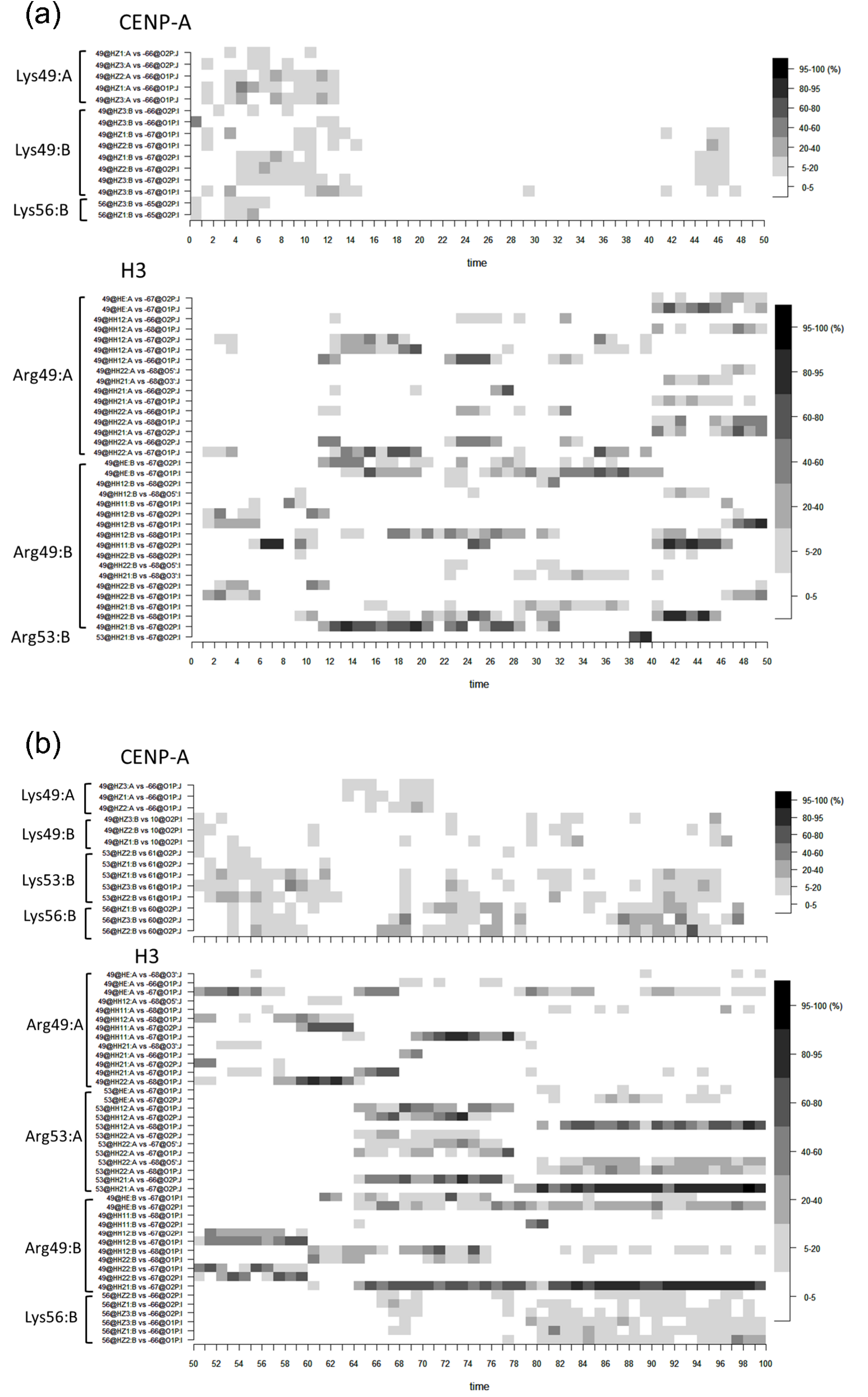

Supplement: S1 Fig — The plots are shown for Lys49, Lys53 and Lys56 in CENP-A from CENP-A-NCP and Arg49, Arg53, and Arg56 in H3 from H3-NCP. (a) During the first 50 ns and (b) during the last 50 ns. Note that only protein atoms that form hydrogen bonds with DNA atoms are listed. (TIF) [file pone.0120635.s001.tif]

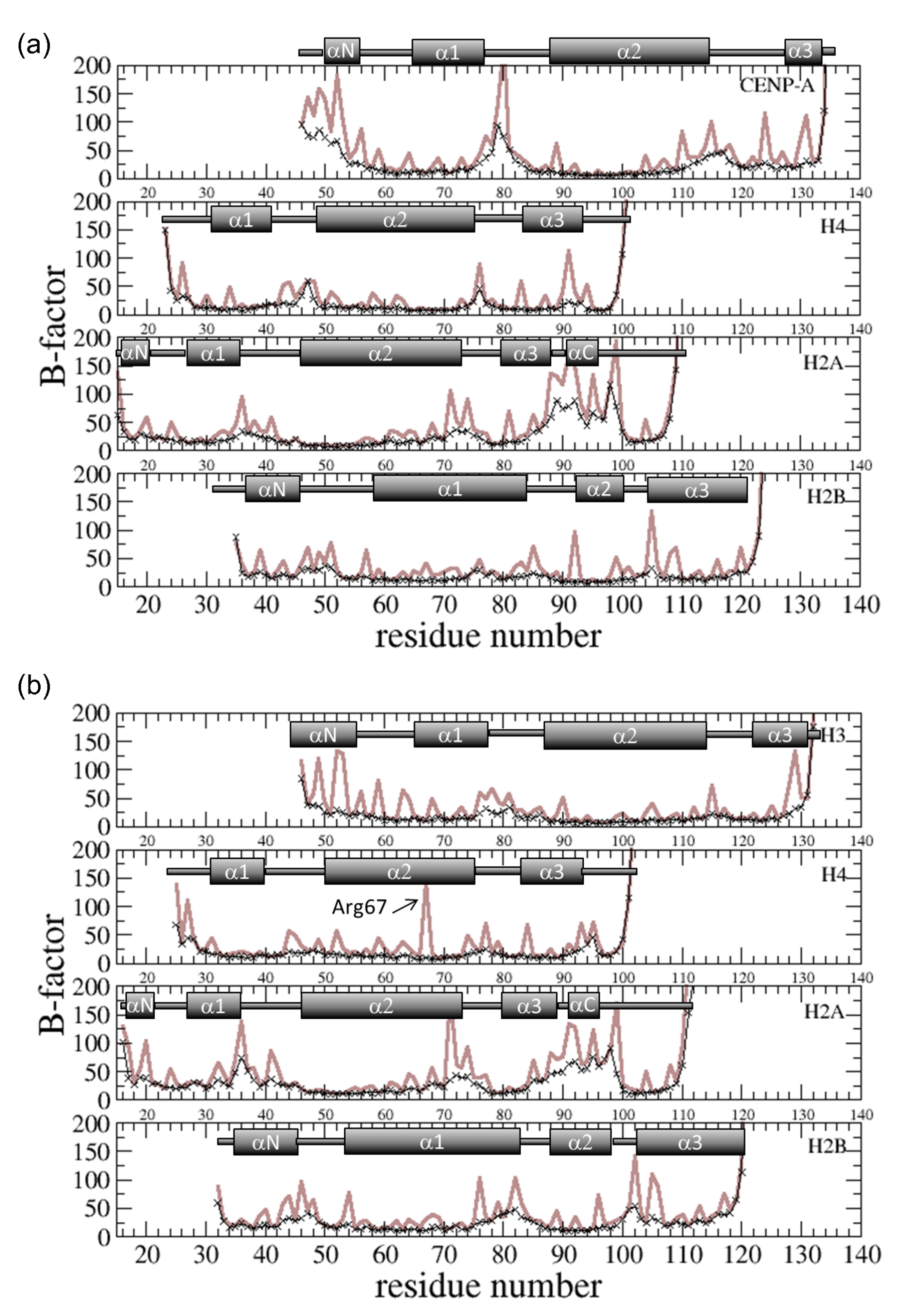

Supplement: S2 Fig — (a) CENP-A-NCP and (b) H3-NCP. The B-factors values of the main chain heavy atoms (black lines) and all heavy atoms (brown lines) were calculated using 100 ns-long MD trajectories. At top of each panel, the secondary structures are shown based on the crystal structures. (TIF) [file pone.0120635.s002.tif]
